# The changing landscape of semiconductor manufacturing: why the health sector should care

**DOI:** 10.3389/frhs.2023.1198501

**Published:** 2023-06-12

**Authors:** Kevin Zhai

**Affiliations:** Department of Medical Education, Weill Cornell Medicine-Qatar, Cornell University, Doha, Qatar

**Keywords:** semiconductors, medical devices, integrated circuits, pure play manufacturer, fabrication

## Abstract

As semiconductor devices become increasingly ubiquitous in healthcare, the health sector has in turn grown highly dependent on the semiconductor industry. This relationship is not always symbiotic, and even mild turbulence in the semiconductor industry has the potential to derail patient care. Here, we introduce semiconductor manufacturing and discuss political and economic forces that will shape the industry for years to come. The uncertain outlook for semiconductors underscores the need for stakeholder collaboration to ensure an adequate supply of semiconductor-utilizing medical devices for the patients of today and tomorrow.

## 1. Introduction

In the digital age, semiconductor devices have become ubiquitous across nearly all industries, and the health sector is no exception. These microelectronics enable diverse technologies ranging from magnetic resonance imaging (MRI) to insulin pumps (1). Patients track their vitals with mobile sensors and clinicians exchange data through computer platforms—which would be inoperable without the semiconductor chips from which computer memory and processing power are derived. In fact, 50% of all medical devices utilize semiconductors (2). Hence, the continued success of modern healthcare is predicated on the health and profitability of the semiconductor industry.

Chip manufacturing has evolved constantly over the past half century, with considerable improvements in consistency, throughput, and efficiency. The economics of modern chip fabrication enable the mass production of affordable consumer- and industry-grade computing devices (3). However, the industry has undergone significant restructuring as political and economic forces have shifted in recent years, threatening the current status quo.

## 2. Semiconductor manufacturing

In the late 1950s, the world of technology was transformed by the invention of the metal oxide semiconductor field effect transistor (MOSFET). Linking of these efficient and scalable transistors with other electrical components on a single Si chip yielded an integrated circuit (IC)—the basis of modern computer processors (4). Development of this technology in the following decades obeyed Moore's Law, which predicts that the number of transistors that can be fit in an IC doubles roughly every 2 years (5). As manufacturing techniques matured, MOSFETs became exponentially smaller, allowing ever smaller ICs to hold ever greater processing power; accordingly, computing devices became smaller and more affordable. Today, the newest Apple iPhone is powered by an IC with 16 billion transistors.

Manufacturing of these ICs, with feature sizes at the nanoscale, requires highly specialized processes and tools. A robust industry centered on IC fabrication has thus emerged and matured, centering on IC manufacturers that operate foundries, known as “fabs,” to mass-produce devices that incorporate ICs. There are two major types of these corporations:

A. Integrated device manufacturers (IDMs) design, produce, and market their own IC devices. Examples include Texas Instruments, Samsung, and Intel.
B. Pure-play manufacturers (PPMs) are contracted to produce IC devices that are designed and marketed by other companies known as fabless semiconductor companies. In this model, the fabless semiconductor company utilizes the PPM as its fab. Examples of PPMs include Taiwan Semiconductor and GlobalFoundries, and examples of fabless semiconductor companies include Apple and Microsoft.

While IDMs and PPMs employ similar processes in their fabs, they differ considerably in their economics; this becomes significant in the context of recent developments in the industry.

## 3. Political and economic considerations

The modern semiconductor industry plays a foundational role in a world dependent on processors of various shapes and sizes. Fabs sit in prominent positions in supply chains for computers, mobile phones, medical devices, and beyond. However, with increasingly complex regulatory hurdles and thinning profit margins, IC manufacturers have struggled to keep pace with burgeoning consumer demand, leading to downstream supply chain difficulties.

As suggested by their business model, PPMs produce a significant proportion of the ICs used in consumer-facing electronic devices. A limited number of PPMs dominate the global market, with a single company, TSMC, holding more than 50% of the market (Table 1). Heavy global reliance on this small contingent of companies increases the likelihood of a catastrophic single-point failure that would impact all sectors of today's digitalized economy; the health sector would certainly not be spared.

**Table 1:** PPMs by global market share from 2019 to 2022 (6).

Corporation	Operating base	Market share
Taiwan Semiconductor Manufacturing Company (TSMC)	Taiwan	50%–60%
United Microelectronics Corporation (UMC)	Taiwan	5%–10%
GlobalFoundries	USA, Singapore	5%–10%
Semiconductor Manufacturing International Corporation (SMIC)	P.R. China	5%–10%

The ongoing worldwide semiconductor and IC shortage, brought about by the COVID-19 pandemic and international political and economic shifts, is an excellent case study that illustrates the health sector's vulnerability. In 2021 and 2022, medical device manufacturers faced increasing difficulties acquiring ICs for use in their devices. A 2022 Deloitte survey found that many of these manufacturers had decreased or halted production of their medical devices as a result, with more than 75% reporting that their healthcare provider customers are utilizing alternative treatment modalities (7). Despite these direct effects on patient care, the impact of the semiconductor shortage has received minimal attention in the medical literature.

Today's shortage of medical devices reveals that even a minor deficit in the semiconductor industry can have far-reaching consequences; only time will tell if patient outcomes are adversely impacted. Based on this, one can only imagine the consequences for healthcare if one of the “Big Four” PPMs were to fail. With three of the four based in Taiwan or the People's Republic of China, the political tensions in the region are of significant concern. Moreover, even in the absence of political undercurrents, economic forces will favor some PPMs over others, as was seen when IBM entirely divested its PPM arm (the Microelectronics Division) in 2015. Similar changes are to be expected in the future; nonetheless, the health sector is unprepared.

An effective remedy for this vulnerability will require collaboration between and investment from the stakeholders in healthcare, industry, and beyond. However, a prerequisite for any solution-seeking efforts is definition of the problem and characterization of its nature and scope. The academic and research community stands to play a key role in this regard, through both retrospective analysis and prospective modeling of potential impact scenarios. A study of the impact of COVID-19 on semiconductor manufacturing and the consequent impact on healthcare, for instance, would be highly pertinent. With the information gleaned from these studies, governments and policy makers would be better equipped to engage healthcare and semiconductor industry leaders in seeking a solution that balances economic and health interests.

## 4. Conclusions and outlook

Semiconductor manufacturers wield undue influence over today's technologically-dependent health sector; though 50% of medical devices utilize semiconductors, these medical devices comprise only 1% of the total market for semiconductors (2). As such, while the semiconductor industry could remain profitable without the health sector, the health sector could not exist in its current form without the semiconductor industry. Leaders in healthcare, with the support of policy makers and researchers, must therefore develop relationships with semiconductor manufacturers and advocate for the health sector as a whole: a reliable supply of life-saving devices such as pacemakers and insulin pumps ought to be non-negotiable.

## Data Availability

The original contributions presented in the study are included in the article, further inquiries can be directed to the corresponding author.
